# Play and Be Happy? Leisure Participation and Quality of Life in School-Aged Children with Cerebral Palsy

**DOI:** 10.1155/2012/387280

**Published:** 2012-08-07

**Authors:** Keiko Shikako-Thomas, Noémi Dahan-Oliel, Michael Shevell, Mary Law, Rena Birnbaum, Peter Rosenbaum, Chantal Poulin, Annette Majnemer

**Affiliations:** ^1^School of Physical and Occupational Therapy, McGill University, Montreal, QC, Canada; ^2^Division of Pediatric Neurology, Montreal Children's Hospital, McGill University Health Centre, 2300 Tupper Street, Room A-509, Montreal, QC, Canada H3H1P3; ^3^School of Rehabilitation Science, McMaster University, Hamilton, ON, Canada; ^4^CanChild Centre for Childhood Disability Research, McMaster University, Hamilton, ON, Canada; ^5^Occupational Therapy, Montreal Children's Hospital, McGill University Health Centre, Montreal, QC, Canada; ^6^Department of Pediatrics, McMaster University, Hamilton, ON, Canada

## Abstract

The objective of this study was to examine the association between leisure participation and quality of life (QoL) in school-age children with cerebral palsy (CP). Leisure participation was assessed using the Children's Assessment of Participation and Enjoyment (CAPE) and QoL using the Pediatric Quality of Life Inventory (PedsQL). Pearson correlation coefficients were calculated to examine the association between CAPE and PedsQL scores, and a multiple linear regression model was used to estimate QoL predictors. Sixty-three children (mean age 9.7 ± 2.1 years; 39 male) in GMFCS levels I–V were included. Intensity of participation in active-physical activities was significantly correlated with both physical (*r* = 0.34, *P* = 0.007) and psychosocial well-being (*r* = 0.31, *P* = 0.01). Intensity and diversity of participation in skill-based activities were negatively correlated with physical well-being (*r* = −0.39, *P* = 0.001, and *r* = −0.41, *P* = 0.001, resp.). Diversity and intensity of participation accounted for 32% (*P* = 0.002) of the variance for physical well-being and 48% (*P* < 0.001) when age and gross motor functioning were added. Meaningful and adapted leisure activities appropriate to the child's skills and preferences may foster QoL.

## 1. Introduction

Cerebral palsy (CP) is a broad term that describes a set of conditions that is associated with major physical impairments and other developmental deficits and arises in the early stages of brain development [1]. CP is the most common type of physical disability affecting children in developed countries [2] with an estimated prevalence of 2.0 to 2.5/1000 children [3]. Despite the nonprogressive nature of the condition, the nature of functional impairments may change as the child develops. As a consequence, evolving limitations in everyday activities may be experienced, with possible impact on the individual's overall health and well-being [4, 5]. The type and distribution of movement disorder in CP may be categorized as spastic, dyskinetic, hypotonic, or mixed. Spastic patterns are the most common and may be further differentiated as diplegia, quadriplegia, hemiplegia, or monoplegia, relating to the limbs involved [6, 7].

Individuals with CP have been an important target population requiring rehabilitation services. The traditional focus of health care services for this population has been primarily directed at rehabilitation interventions that address the underlying motor and other developmental impairments, such as abnormal muscle tone, decreased attention span, poor dexterity, or difficulties with perceptual concepts, as well as limitations in essential daily self-care skills and mobility. In the last decade, however, increasing interest has been attributed to the quality of life (QoL) and participation of children with CP. Researchers and clinicians are concerned with the extent to which children with CP have the opportunity to be involved and enjoy leisure activities at home and in the community and the extent to which they report a good QoL [8, 9].

QoL is a broad concept encompassing many components of overall health and well-being (e.g., physical, psychosocial, economic, and cultural). It is influenced by the context of the culture and value systems in which the individual lives and relates to the individual's goals, expectations, standards, and concerns [10]. The QoL of children who live with a neurological condition can be impacted at different levels, including physical (e.g., physical health, independence in basic functional activities), psychological (e.g., mental status, positive self-perception), and psychosocial dimensions (e.g., forming friendships, leisure time, finding a partner) [11]. Identifying the factors that are associated with better or poorer QoL is important [12] as this can be used to guide program planning and the allocation of resources, thus optimizing the well-being of these children. 

Studies have shown that individuals with disabilities do not necessarily have a lower QoL and a diminished perception of their well-being or general dissatisfaction with life [13, 14]. Recent studies are exploring a variety of factors that may contribute to a good QoL in children with neurodevelopmental disabilities; however, most studies explore aspects related to body function and activity limitations.

Participation in leisure activities includes participation in sports, arts, entertainment, social, self-improvement, and religious activities [15]. Engaging in leisure activities may be influenced by the child's personal factors, environmental factors, and underlying health condition [15]. By participating in leisure activities, children may develop competencies, achieve mental and physical health, gain an understanding of their strengths and abilities, and form lasting meaningful friendships and relationships. It is through participating that children make contributions to their community, learn about themselves and the expectations of society, and develop skills needed to become successful and autonomous in their home, school, and community [16, 17]. For these reasons, participation is increasingly considered as one of the primary aims of pediatric rehabilitation and is believed to contribute to child health, development, and QoL [16, 18]. Researchers have shown that multiple factors may contribute to a perceived good QoL in children with physical disabilities [17], including the child's participation in a variety of leisure activities [19]. 

Participation in leisure is an objective, tangible outcome that can be incorporated and measured in rehabilitation and health programs, while QoL is a subjective outcome [20]. Although frequently assumed, the association between leisure participation and QoL has not been clearly delineated [21]. The objective of this study was to examine the association between involvement in leisure activities and QoL in children between 6 to 12 years of age with CP.

## 2. Methods

### 2.1. Population

This study used a cross-sectional design involving a historical cohort of children with CP seen by a single neurologist over a 10-year period (1991–2001) in a variety of settings (private office, hospital, neonatal clinic, suburban private clinic). This sample has been described previously [22–25]. This sample is representative of children with CP from a local community who are routinely sent to a pediatric neurologist for diagnosis, etiological determination, and referral for rehabilitation services. Exclusion criteria were children presenting progressive disorders, disorders of noncerebral origin, and specific syndromes. Parents who could not easily read or converse in English or French were excluded from participation.

A total of 63 children and their parents completed the leisure participation and QoL questionnaires. Twenty-one participants who could not actively participate in the completion of the leisure participation questionnaire were excluded. Those participants were children with more severe cognitive impairment and therefore who could not actively contribute to the completion of these self-report questionnaires. These children were more likely to be level IV or V GMFCS. Demographic and clinical characteristics of the participants and also of the children who could not complete the questionnaires (nonparticipants) are described in Table 1. 

### 2.2. Procedures

Scientific and ethical approval was obtained through the McGill University Health Centre, Montreal Children's Hospital's institutional review board. Information letters describing the study were sent to parents of children with a diagnosis of CP between the ages of 6 and 12 years. Informed signed consent was obtained from a parent, and assent was obtained from children when feasible. A 3-hour visit was scheduled at the Montreal Children's Hospital to conduct required assessments. Participants completed a series of developmental evaluations administered by a neurologist and a physical therapist and/or occupational therapist. As part of this study, the neurologist assessed the level of gross motor function for each participant using the 5-level Gross Motor Function Classification System (GMFCS) [26]. The Gross Motor Function Measure (GMFM-66) [27] was administered by a physical or occupational therapist during the visit. Parents (and children when feasible) completed questionnaires assessing leisure participation and QoL and also completed a demographic questionnaire.

### 2.3. Outcome Measures

The Pediatric Quality of Life Inventory (PedsQL) Generic Core Scales [28] were used to assess quality of life in the physical, emotional, social, and school domains. This questionnaire includes 23 items, and the parent proxy-report version was used in this study. The child-report version was also used when feasible, but, due to smaller numbers, the parent report was used for analysis in this paper due to the small sample of children who were able to complete the child version independently. Individual domain scores were calculated, as well as psychosocial and physical summary scores. The psychosocial well-being summary score represents the way the child feels about their social life, their school functioning, and their emotional well-being. The physical summary score represents the ease in which one can get around and do basic activities, without pain while maintaining a good energy level. Internal consistency reliability of the PedsQL is excellent (alphas > 0.90). The validity of the PedsQL Generic Core Scales has been demonstrated through known groups comparisons and correlations with other measures of disease burden [28]. 

The Children's Assessment of Participation and Enjoyment (CAPE) [29] was used to assess participation in leisure activities. This 55-item child self-report questionnaire was designed for children and youth with and without disabilities between the ages of 6 and 21 years. It provides information on five dimensions of participation (diversity, intensity, where, with whom, and enjoyment) and five types of activities (recreational, active-physical, social, skill-based, and self-improvement). Current evidence supports the tool's validity, and reliability is adequate [30]. Parental assistance was sought when children had difficulty completing this questionnaire; however, children actively contributed in the completion of the CAPE. For the purposes of this study, intensity (how often does the child participate in each activity) and diversity (how many different activities a child engages in) scores in the five different activity types were used. 

### 2.4. Data Analysis

Descriptive statistics were used to describe the sample. Participants (children with CP) who could not actively participate in completion of the CAPE were excluded from analysis. Pearson correlation coefficients were computed to examine the association between leisure participation (CAPE) and QoL (PedsQL). A multiple linear regression model was estimated with QoL (psychosocial and physical summary scores) as the dependent variable and leisure participation as the independent variable. Models were also derived including age and level of motor function using GMFM scores. 

Diagnostic tests were used to check for violation of the assumptions inherent in linear regression models. Due to the lack of evidence on specific leisure domains that may predict QoL, forced entry regression was used to determine the significant predictors of psychosocial and physical QoL. This method is likely not influenced by random variation in the data and is therefore appropriate for using theory testing [31].

## 3. Results

 When reporting their child's QoL, about half of the parents reported that their child had low well-being (more than one standard deviation below normative means) in the physical and psychosocial well-being domains. Well-being in emotional functioning and school functioning was within normative means for about 60% of the children as reported by their parents (Table 2). Children participated in a variety of leisure activities, but mostly in informal activities. Intensity scores measure how often the child has engaged in a given activity in the past four months (ranging from once in the past four months to everyday or more). The intensity of recreational activities (e.g., doing crafts) was higher than the other four activity domains, followed by social activities (e.g., talking on the phone) and self-improvement activities (e.g., reading) (Table 3). A detailed description of participation in leisure activities and QoL for this population has been published elsewhere [22–24].

The relationship between involvement in leisure activities (i.e., diversity and intensity of participation in activities) and parent-proxy report of child's QoL was tested. Intensity of participation, but not diversity, in active-physical activities such as team sports, bicycling, water and snow sports, and other individual physical activities was significantly correlated with physical well-being (*r* = 0.34, *P* = 0.007). Intensity and diversity of involvement in skill-based activities, that is, of activities such as dancing, arts, and music classes done with an instructor, were negatively correlated with physical well-being (*r* = − 0.39, *P* = 0.001 for intensity and *r* = − 0.41, *P* = 0.001 for diversity, resp.). Intensity of participation in active-physical activities also accounted for better psychosocial well-being (*r* = 0.31, *P* = 0.01) of children with CP. 

In a multiple regression model, diversity and intensity of participation in five domains of leisure accounted for 32% (*P* = 0.002) of the variation in physical well-being. However, when age and gross motor functioning (GMFM score) were included in the model, this value increased to 48% (*P* < 0.001) of the variance in the physical well-being domain (Table 4). None of the multiple regression models was significant for psychosocial well-being.

## 4. Discussion

This study describes the association between participation in leisure activities and QoL in school-aged children with CP. Results indicate a positive association between engagement in physical activities and both physical and psychosocial well-being. These findings suggest that school-aged children with CP who participate more actively in physical activities get around more easily to do basic activities, without pain and with a good energy level, and feel better about their social life, school functioning, and their emotions, according to parent-report. Inversely, it is possible that children who generally feel better in these domains naturally participate more in active physical activities. Skill-based activities and physical well-being were negatively related, suggesting that other factors may play a role in this relationship. For instance, motor functioning alone accounted for a high variance in the outcome variable in the multivariate model. Indeed, child attributes such as the severity of motor dysfunction, age, and gender were previously described as important predictors of physical well-being, a component of a child's QoL [22, 32, 33]. Studies have shown that, with increasing age, children tend to decrease their participation in out of school leisure activities [17]; therefore, it is important to motivate them to maintain and increase their activity level as they develop. 

Other personal and environmental factors may mediate the association between leisure participation and QoL. For instance, children and families who do not have the environmental supports and adaptations they require may not be able to participate in leisure activities of their own choosing, which is related to the child's QoL. Furthermore, children with more severe motor limitations often attend segregated school environments where they are exposed to more intense rehabilitation services and adapted leisure activities as compared to children in an integrated school setting. Children in special schools may have the opportunity to participate in activities such as adapted horseback riding, adapted arts, and swimming programs. Half of the parents of school-aged children with CP in our study reported their children as having poorer physical well-being (<1 SD of the normative mean) compared to parents of typically developing peers. This finding is probably related to their motor limitations, which may also explain our result that higher levels of participation in skill-based activities were associated with lower physical well-being. Skill-based activities (e.g., dance lessons, karate lessons) may be challenging for children with disabilities, especially if the demands of the particular task are not adapted to the child's capabilities. Families should be assisted in choosing appropriate and meaningful activities and learning about available adapted sports and recreation programs in their community to foster a sense of mastery and competence. In this regard, it is interesting to observe that children in adapted or segregated schools may have more opportunities to participate in skill-based activities as adapted skill-based activities may be provided by the school or referred to by professionals in the school, in spite of their higher motor limitations.

Physical activity has substantial benefits to the health and well-being of children and adolescents. Active-physical pursuits are known to improve cardiovascular and emotional health, motivating children to stay physically active and contributing to a sense of well-being [34, 35]. Participation in leisure activities also fosters friendships and other social relationships, often creating a social support network that may contribute to the individual's overall well-being [19]. In congruence with the findings of our study, a recent study with 120 school-aged children with CP using different measurement tools reported a significant relationship between the intensity of participation in leisure activities with physical well-being, emotional well-being, and social support and peers [32]. This particular study measured leisure participation more globally; however, we applied a measure with five subscales of different types of leisure activities. Our study indicates that it is the active-physical leisure activities specifically that are most strongly related to aspects of QoL.

Associations between emotional and psychosocial well-being and participation in leisure have also been reported when children engage in everyday activities or social activities [32, 36]. Qualitative studies have shown how children and adolescents with disabilities place a high value on participating in leisure activities. Youth with CP and their parents reported that participation in chosen and enjoyable activities has a positive impact on their well-being [37–42]. A recent analysis of the association between leisure participation and QoL [19] in children with neurodevelopmental disabilities found evidence from quantitative, qualitative, and mixed-methods studies supporting the relationship between participation in leisure and different domains of QoL [32, 33, 35–38]. Similarly to our study, others have found a positive association between engagement in active physical activities and physical well-being in children and youth with CP [32, 35, 43]. However, the measurement of both constructs (QoL and participation) is very variable across studies, limiting interpretability for clinical practice. Our study, however, is the first to report on the relationship between psychosocial and physical domains of QoL, specifically with respect to leisure participation in school-age children with CP.

## 5. Limitations

Measurement issues should be taken into consideration when analyzing findings of this study. The CAPE measures the activities that the child actually does (i.e., performance), which may or may not be in line with their preferences or personal choices. A strong linear relationship between CAPE diversity and intensity scores may account for a limited capacity of the model to represent the sample and limit the size of regression coefficients in the regression models. A moderate correlation between levels of actual involvement in active-physical pursuits with the level of preferences for these types of activities was previously shown [25], implying that children may be participating in activities that they do not necessarily enjoy. Programs and interventions should consider the preferences of children and families in order to individually tailor the level of engagement in activities to their preferences (i.e., through adapted programs or those that have no environmental barriers) and thus foster better overall well-being. Parental report was used for the QoL measure in this study. Interrater reliability between parental and child report of QoL was previously described for this sample and shown to be very good for the physical domain (ICC = 0.72, *P* < 0.0001), but less for the psychosocial aspect of QoL (ICC = 0.54, *P* < 0.0001) [24]. Unfortunately, 21 children were either too young or had cognitive and/or language deficits that limited their ability to self-report. Whereas the CAPE can be completed with the help of an interviewer and uses pictorial and verbal depiction of the items, the self-report version of the PedsQL requires more ability to be completed. Therefore, we chose to use the PedsQL parent-report version in the analysis. This is a frequently reported challenge in measurement of subjective constructs in children with complex disabilities. Children may place a higher value than their parents on engagement in leisure activities, and an association between the child's self-report leisure participation and their QoL may be stronger than findings using parental report. The fact that only children who could self-report were considered in this study clearly indicates a need to explore other measurement alternatives for children with more severe impairments in order to promote QoL for children across all severity levels. Due to the cross-sectional design of this study, a causal relationship between leisure participation and QoL cannot be inferred. For instance, children who participate in more leisure activities may experience better QoL than children who participate in fewer or less frequent activities. The opposite may also be true, such that children who experience a higher sense of physical and psychosocial well-being may engage in more leisure activities. 

## 6. Conclusion

Our findings indicate that leisure participation in active-physical activities is positively associated with both physical and psychosocial well-being in school-aged children with CP. Meaningful and adapted leisure activities appropriate to the child's skills and preferences may foster QoL. Further studies are needed to explore the temporal and possible causal connections between these aspects of children's lives. QoL and participation are important complex, multidimensional conceptual constructs that encompass many subjective aspects, including individual preferences, the influence of environmental factors, but also objective aspects such as the availability, accessibility, and affordability of preferred activities, the school setting, and the family's involvement in specific activities. Assessment of both constructs and interventions aiming to improve the QoL of children with CP should include the promotion of meaningful and adapted leisure activities appropriate to the child's skills and preferences, especially active-physical activities.

## Figures and Tables

**Table 1:** Characteristics of participants.

*N* = 63		Study participants (*n* = 63)	Nonparticipants (*n* = 21)
Mean age		9.7 ± 2.1 years	8.69 ± 1.9 years

Gender		62% male	52% male

Gross Motor Function Classification System (GMFCS)	Level I	59%	10%
	Level II	22%	—
	Level III–V	18%	90%

CP distribution	Spastic quadriplegia	24%	61%
	Spastic hemiplegia	35%	9.5%
	Spastic diplegia	24%	9.5%
	Other	17%	4.8%

School setting	Regular school	56%	24%
	Special school	44%	76%

Rehabilitation services	Received rehabilitation services in the past 6 months	84%	100%

Socioeconomic status (combined household income before taxes)	0–$19,000	12%	13%
	$20,000–$39,000	27%	27%
	$40,000–$59,000	21%	20%
	$60,000–$79,000	14%	27%
	$80,000+	26%	13%

**Table 2:** Performance on PedsQL domains.

PedsQL parent-report *N* = 63	Score per domain mean (range)	Abnormal mean score (<1 SD from normative data)
Emotional functioning	68.5 (40–100)	39.7%
Social functioning	58.3 (25–100)	50.8%
School functioning	62.9 (25–100)	39.7%
Physical functioning	62.1 (12.5–100)	50.8%

Physical summary score	62.1 (12.5–100)	50.8%
Psychosocial summary score	63.2 (35–100)	55.6%

**Table 3:** Mean scores of domains of participation in leisure activities as assessed by the CAPE.

Domain of participation *N* = 63	Diversity mean (range)	Frequency mean (range)	Enjoyment mean (range)
Recreational activities	9.34 (5–12)	4.10 (1.6–6)	4.28 (2.9–5.1)
Active physical activities	3.03 (0–7)	1.68 (0–4.33)	4.49 (2.6–5.3)
Social activities	6.87 (4–9)	3.12 (1.5–4.7)	4.37 (2.7–5.3)
Skill-based activities	2.8 (0–6)	1.45 (0–3.8)	4.12 (1–5)
Self-improvement activities	5.58 (1–9)	2.82 (0.6–5.6)	3.5 (1.5–5)

Formal activities	3.87 (0–9)	1.28 (0–3)	4.06 (0–5)
Informal activities	23.77 (13–31)	3.3 (1.8–4.8)	4.16 (3–5)

Scoring: Diversity (number of different activities) = number of activities involved in compared to total number of activities. Intensity (how often) = (1) 1x/4 months, (2) 2x/4 months, (3) 1x/month, (4) 2-3x/month, (5) f1x/week, (6) 2-3x/week, (7) daily. Enjoyment (how much do you enjoy) = (1) not at all, (2) somewhat, sort of, (3) pretty much, (4) very much, (5) love it.

**Table 4:** Best predictive models of physical well-being (PedsQL).

Model variables (*N* = 60)	*R* ^2^ (*P*)	Best predictors beta (*P* value)
Diversity of leisure activities (all domains)	0.23 (<0.005)	Skill-based activities
		−0.49 (<0.001)
Diversity and intensity of leisure activities, age, and motor functioning	0.48 (<0.001)	GMFM-66
		0.61 (<0.001)
